# Health Care Professionals’ Perspectives of Socially Assistive Robots in Health Care Settings: Systematic Review

**DOI:** 10.2196/79634

**Published:** 2025-10-09

**Authors:** Yun Hsuan Lee, Fang Yu Hsu, Angela Shin-Yu Lien

**Affiliations:** 1 Graduate Institute of Nursing College of Medicine Chang Gung University Taoyuan Taiwan; 2 Department of Nursing Chang Gung Memorial Hospital Linko Branch Taoyuan Taiwan; 3 School of Nursing College of Medicine Chang Gung University Taoyuan Taiwan; 4 Department of Internal Medicine Division of Endocrinology and Metabolism Chang Gung Memorial Hospital Linko Branch Taoyuan Taiwan

**Keywords:** health care professionals, health care providers, health care workers, perspectives, robot, robotic, socially assistive robots, systematic review

## Abstract

**Background:**

Health care professionals (HCPs) are key stakeholders whose acceptance, preparedness, and ethical considerations influence the integration of socially assistive robots (SARs). This review explores HCPs’ perspectives on SARs integration into clinical practice. While previous research has focused on patient outcomes, ethical considerations, or general SARs deployment, limited evidence exists on how HCPs perceive, engage with, and address SARs implementation challenges.

**Objective:**

This study aims to systematically analyze HCPs’ perspectives on the clinical implementation of SARs, including acceptance, challenges, barriers, educational needs, and ethical concerns.

**Methods:**

Following PRISMA (Preferred Reporting Items for Systematic Reviews and Meta-Analyses) 2020 guidelines, we searched 13 databases (PubMed, Cochrane Library, Scopus, IEEE Xplore, ScienceDirect, CINAHL, Epistemonikos, MEDLINE [OVID], Web of Science, Embase, UpToDate, CEPS, and ProQuest Dissertations and Theses Global), with the final search on July 29, 2025. Eligible studies involved research with HCPs, examining their attitudes, perceptions, acceptance, or willingness to use SARs through qualitative, quantitative, or mixed methods designs. Studies were excluded if they focused on non-HCP populations, did not primarily investigate SARs, or lacked original data. Risk of bias was assessed with the Mixed Methods Appraisal Tool. Findings were synthesized thematically and mapped to the Unified Theory of Acceptance and Use of Technology (UTAUT) framework.

**Results:**

A total of 15 studies (6 qualitative, 5 quantitative, and 4 mixed methods) involving 3166 HCPs across 10 countries were included. Participants were predominantly nurses and midwives (1960/3166, 61.9%), female (2618/3166, 82.7%), and based in hospital and long-term care settings across Europe (1709/3166, 54%) and Asia (1266/3166, 40%). Study quality was generally moderate, with 1 high-quality and 2 low-quality studies. Within the UTAUT framework, HCPs anticipated benefits of SARs in workload reduction, enhanced care efficiency, and improved patient well-being. However, they expressed concerns about technological reliability, maintenance requirements, role clarity, and professional identity. Acceptance was generally favorable but varied by profession, workplace, and relational attributes. Training needs, usability, and design were critical adoption determinants. Ethical concerns centered on privacy, informed consent, and equitable access.

**Conclusions:**

Although evidence was limited due to moderate methodological quality, small sample size, self-developed instruments, and inconsistent reporting, which constrain generalizability, this review synthesis suggests that HCPs perceive SARs as beneficial for reducing workload, enhancing efficiency, and supporting patient well-being. However, there are also concerns regarding technological reliability, ethical challenges, and role boundaries. Acceptance is facilitated by ethical literacy, training, and organizational readiness. Interdisciplinary strategies that integrate educational, ethical, and structural considerations to promote the adoption of responsible SARs in health care.

**Trial Registration:**

PROSPERO CRD420251079714; https://www.crd.york.ac.uk/PROSPERO/view/CRD420251079714

## Introduction

The global health care system is facing critical workforce shortages, underscoring the urgency of exploring innovative technologies to sustain health care delivery. The World Health Organization estimated a global deficit of approximately 4.5 million nurses by 2030 [1]. This pressing context has drawn increasing attention to digital technologies, including artificial intelligence (AI) and robotics, as potential strategies to enhance efficiency and maintain quality of care [1]. Among these innovations, socially assistive robots (SARs), robots designed to provide support through social interaction rather than physical contact, have emerged as promising tools to improve workflow efficiency and patient well-being [2,3]. To ensure conceptual clarity, we consistently use the term SARs in this review, as naming conventions in health care technology can shape how such innovations are understood and implemented [4].

SARs are designed to facilitate social interaction and engagement by performing tasks such as companionship, reminders, guidance, and cognitive stimulation. Evidence suggests that SARs can provide emotional comfort, rehabilitation support, and enhance patient participation across various health care settings, ranging from long-term care facilities to pediatric hospitals [5-10].

Health care professionals (HCPs) are crucial stakeholders in the sustainable implementation of SARs [11]. However, their perspectives remain insufficiently examined. Previous systematic reviews and empirical studies have primarily focused on patient outcomes from SARs use [8,9], ethical considerations within clinical contexts [12], or SARs deployment in specific health care settings [6] and pediatric environments [7]. Papadopoulos et al [13] mapped SARs deployment globally without analyzing HCPs' perspectives. Similarly, Aymerich-Franch and Ferrer [14] reviewed 19 studies on nurses’ and social care workers’ views, reporting mixed attitudes on utility, safety, and privacy, but the review was limited in scope and lacked theoretical framing. Relatedly, Ala’a [15] synthesized factors influencing HCPs’ acceptance of AI-based technologies, identifying perceived usefulness, ease of use, and behavioral intention as key determinants, while Lambert et al [16] highlighted barriers such as fear of reduced autonomy and challenges in workflow integration, alongside facilitators such as training. These studies have investigated the broader application of AI and robotics in health care or HCPs’ acceptance of AI technologies more generally. Although these areas provide valuable insights, they do not provide a comprehensive understanding of how HCPs perceive and engage in implementing SARs.

This gap is significant because HCPs’ acceptance and preparedness are key determinants of successful SARs integration into clinical practice. Furthermore, education, training, and competencies are critical for technology adoption, as emphasized in previous research [5]. Addressing this overlooked dimension is therefore essential for advancing both the theoretical and practical development of SARs in health care.

The innovation of this study lies in its comprehensive and practice-oriented approach. Moving beyond examining only HCPs’ acceptance or focusing on the perspective of a single professional group. By situating these factors within the broader context of clinical practice, this study introduced a novel, practice-oriented perspective. Specifically, it emphasizes that the successful deployment of SARs requires strategic educational interventions and ethical frameworks to mitigate real-world challenges.

This systematic review used the Unified Theory of Acceptance and Use of Technology (UTAUT) framework to synthesize evidence from diverse research designs, offering a comprehensive analysis of HCPs’ perspectives on SARs in clinical practice. It integrates HCPs’ viewpoints and addresses the challenges, barriers, educational needs, and ethical considerations related to SARs implementation. By consolidating fragmented insights, this review identifies critical gaps in the current knowledge and delineates future research directions for the effective integration of SARs into clinical settings.

## Methods

### Design

This systematic review was registered in the International Prospective Register of Systematic Reviews (PROSPERO; registration CRD420251079714). This review was conducted in accordance with the PRISMA (Preferred Reporting Items for Systematic Reviews and Meta-Analyses) 2020 guidelines [17]. All processes were independently evaluated by 2 reviewers, and any disagreements were resolved by discussion with the corresponding author.

### Eligibility Criteria

The inclusion criteria for the studies were as follows: studies must include HCPs as participants; examine all related perspectives, such as attitudes, perceptions, acceptance, views, or willingness to use SARs; use qualitative, quantitative, or mixed methods research designs; and include original empirical studies involving human participants. Studies were excluded if they focused on non-HCP populations, did not primarily investigate SARs, or were not original research. The eligibility criteria for the inclusion and exclusion of studies are detailed in Textbox 1.

**The eligibility criteria for inclusion and exclusion.**

**Inclusion criteria**

- health care professionals (HCPs)
- Attitudes, perceptions, views, acceptance, or willingness
- Original research
- Primarily investigate socially assistive robots (SARs)

**Exclusion criteria**

- Non-HCP populations
- Without relevant outcomes
- Not original research
- Did not investigate SARs

### Information Source

A comprehensive literature search was conducted across 13 databases, with the final search completed on July 29, 2025. The following databases were systematically searched: PubMed, Cochrane Library, Scopus, IEEE Xplore, ScienceDirect, CINAHL with Full Text (EBSCO), Epistemonikos, MEDLINE (OVID), Web of Science, Embase, UpToDate, and CEPS. In addition, ProQuest Dissertations and Theses Global were reviewed to capture relevant gray literature.

### Search Strategy

Search strategies were formulated using the Population, Intervention, Comparison, and Outcome (PICO) framework and were tailored for each database. A combination of MeSH (Medical Subject Headings) and free-text terms was used along with Boolean operators. The keywords incorporated “Healthcare Professionals,” “Socially Assistive Robot,” “Acceptance,” “Perspective,” “Attitude,” “Perception,” “Views,” and “Healthcare Setting.” Overall, 2 authors independently reviewed and validated the search process to ensure its accuracy and comprehensiveness. The complete search strategy for all databases is provided in Table S1 in Multimedia Appendix 1.

### Study Selection

The 2 reviewers independently performed the identification, screening, eligibility assessment, and inclusion stages. The records were sourced from database searches and supplementary sources, and duplicates were removed using EndNote (version 21; Clarivate). Titles and abstracts were screened to exclude irrelevant studies, and the full texts of potentially eligible articles were subsequently evaluated. Reasons for exclusion at the full-text stage were documented, and any disagreements were resolved through consultation with the corresponding author to reach a final decision. Studies that satisfied all inclusion criteria were included in the final synthesis. The overall study selection process is depicted in the PRISMA flow diagram.

### Collection Process

Data extraction was independently conducted by 2 reviewers. Disagreements were resolved through discussion with the corresponding author to reach a consensus. In instances in which clarification was necessary, the authors of the original studies were contacted. The extracted variables included study details (author, year, and country), participant information (professional type, workplace, and number of HCPs), study purpose, methodology, and reported outcomes related to acceptance, attitudes, perceptions, or willingness to use SARs.

### Quality Assessment

The quality of all included studies was evaluated using the Mixed Methods Appraisal Tool (MMAT) [18]. This process involved a systematic assessment of the risk of bias across 5 domains: selection bias, measurement bias, response bias, confounding bias, and reporting bias, with all evaluations thoroughly documented. The 2 reviewers independently conducted the appraisals, and any discrepancies were resolved through consultation with the corresponding author to achieve consensus. The methodological quality of each study was quantified using a scoring system ranging from 0 to 5 points [16], with criteria rated as “Yes” (1 point), “Unclear” (0.5 points), or “No” (0 points). Studies identified as having a moderate risk of bias were penalized by 1 point, whereas those with high risk of bias were penalized by 2 points. Based on the final scores, studies were classified into 3 levels of quality: high quality (4.5-5 points), moderate quality (3-4 points), and low quality (0-2.5 points).

During synthesis, study quality was explicitly considered; findings from high-quality studies were assigned greater interpretive weight, whereas results from low-quality studies were reported with caution and were acknowledged as less reliable. Potential confounders, including demographic and contextual variables, were identified and discussed to avoid overgeneralization. A narrative synthesis approach was used to integrate heterogeneous study designs while simultaneously recognizing methodological constraints. Transparency was ensured by reporting the distribution of the study quality (low, moderate, and high) and highlighting areas where insufficient rigor may have influenced the evidence. Collectively, these procedures minimized the influence of bias, strengthened validity, and supported a balanced and cautious interpretation of the findings.

### Data Synthesis and Interpretation

Qualitative and quantitative data were analyzed independently before integration. For the qualitative strand, data extracted from the included studies were synthesized using thematic analysis. The material was systematically extracted, inductively coded, and organized into categories, with themes refined iteratively through discussion until consensus was achieved. Quantitative findings were aggregated and summarized descriptively. Integration of the 2 strands followed a convergent parallel approach, whereby qualitative and quantitative results were analyzed separately but compared concurrently through narrative integration. This allowed for the identification of areas of convergence, divergence, and contradiction across data types. The integrated findings are presented in the Synthesis section and subsequently mapped onto the UTAUT theoretical framework [19] according to its respective dimensions.

## Results

### Study Selection

**Figure 1:**
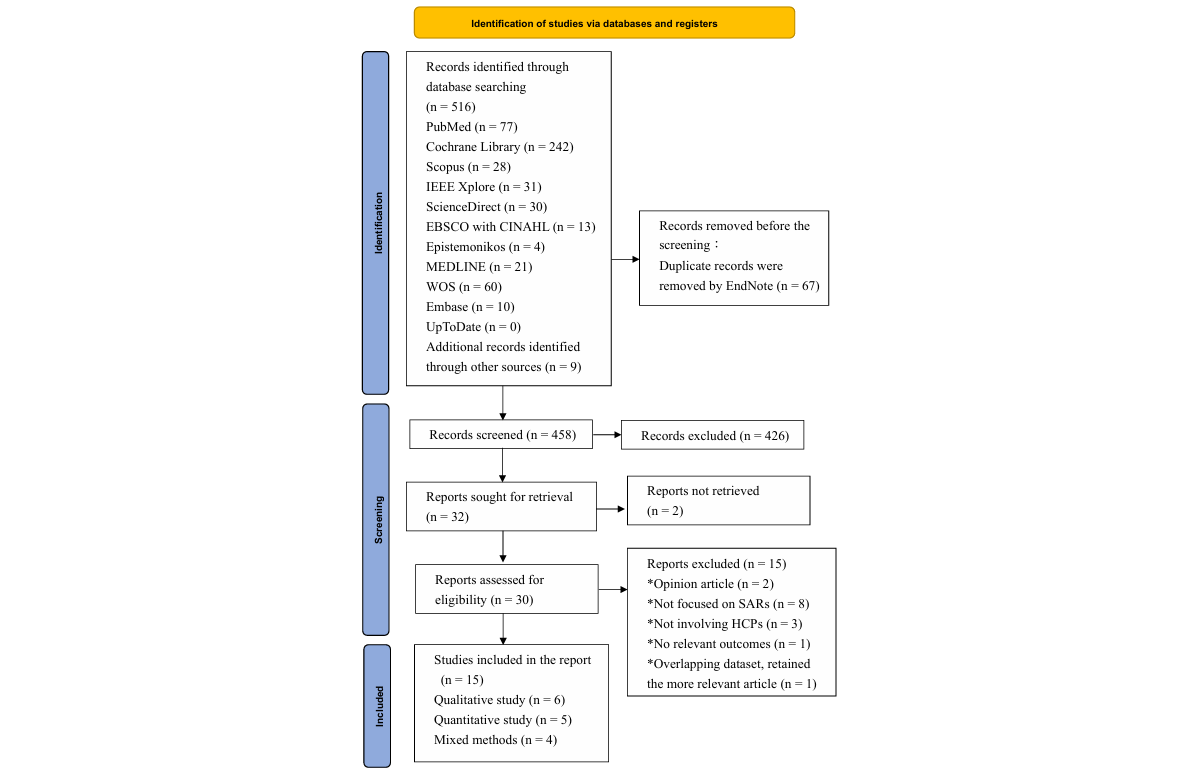
PRISMA flow diagram. HCP: health care professional; SAR: socially assistive robot; WOS: Web of Science.

A total of 516 studies were retrieved from 11 electronic databases, including PubMed, Cochrane Library, Scopus, IEEE Xplore, ScienceDirect, CINAHL, Epistemonikos Database, MEDLINE, Web of Science, Embase, and UpToDate, with an additional 9 studies identified through manual searching as of July 29, 2025. Following the removal of 67 duplicate entries, 458 records were screened by 2 independent reviewers. Of these, 426 were excluded based on the title and abstract that did not meet the eligibility criteria. Full-text versions of 32 articles were sought, of which 2 articles were unavailable for retrieval. The remaining 30 full-text articles were evaluated against the inclusion and exclusion criteria, resulting in the exclusion of 15 studies. Ultimately, 15 studies were included: 6 qualitative, 5 quantitative, and 4 mixed methods studies (Figure 1). The reasons for exclusion included the type of study (opinion article), lack of examination of SARs, absence of focus on HCPs, or failure to address relevant outcomes (Table S2 in Multimedia Appendix 1).

### Study Characteristics

This systematic review encompassed 15 studies conducted in diverse countries, including Israel, England, Colombia, Taiwan, Egypt, Canada, South Korea, Australia, Slovenia, Singapore, and France. These studies investigated HCPs’ perceptions, acceptance, and attitudes toward the implementation of SARs in health care environments. Collectively, the study involved 3166 HCPs with individual study sample sizes ranging from 10 to 1284 participants. HCPs represented a broad spectrum of professions, with the majority being nurses and midwives (1960/3166, 61.9%). The primary settings for these professionals were hospitals (1688/3166, 53.3%) and primary care facilities and nursing homes (845/3166, 26.7%). Most participants were female (2618/3166, 82.7%) and originated from Europe (1709/3166, 54%) and Asia (1266/3166, 40%; Table 1). Furthermore, among the 15 studies included in this review, 8 studies involved participants with no prior direct experience with SARs [20-27]. In 2 studies, participants were shown videos related to SARs before engaging in research activities [28,29]. A total of 5 studies involved direct experience with the actual use of SARs [30-33].

In reporting the results, we explicitly integrated both qualitative and quantitative evidence under the UTAUT framework. To enhance transparency and rigor, the findings were contextualized according to the methodological quality of the contributing studies, as assessed by the MMAT. Subgroup differences (eg, professional role, setting, region, sex, or educational background) were reported, and these were highlighted to provide more targeted insights. Throughout the synthesis, conclusions from higher-quality studies are presented with greater evidentiary weight, whereas results from lower-quality studies are interpreted with appropriate caution.

**Table 1:** Characteristics of the included studies.

Author (year), country	Aim	Study design	HCPs^a^			Outcomes		Quality of study (score)
			Health care setting	Number of professional roles				
Bar-On et al (2023) [20], Israel	Explored the perception of SARs^b^ and the concerns regarding using SARs with PD^c^ patients.	Qualitative	Rehabilitation facilities	n=12 (nurses=3, therapist=8, doctor=1)	SARs were positive to patients, but also concerned that SARs might replace their work.		Moderate (4)	
Bradwell et al (2021) [30], United Kingdom	Explore the acceptability of real-world interaction with SARs.	Qualitative	Domiciliary care, residential care, primary and secondary care, pharmacists, and the mental health unit	n=223 (health and social care professionals=108, others=115)	The potential of SARs with a positive attitude.		Moderate (3)	
Casas et al (2019) [31], Colombia	Explore the attitudes and perceptions of SARs during the cardiac rehabilitation program.	Multimethod	Cardiac rehabilitation unit	n=15 (nurses=3, therapists=2, doctors=10)	Most HCPs consider that the SARs are a useful tool.		Moderate (3)	
Chen et al (2020) [21], Taiwan	Explored attitudes toward using SARs for older adults in long-term care.	Cross-sectional	Nursing home, residential aged care, and rehabilitation ward	n=416 (nurses=313, therapists=8, doctors=3, others=92)	Generally positive attitudes to SARs, especially in nursing homes.		High (4.5)	
El-Gazar et al (2024) [22], Egypt	Explore the behavioral intentions of nurses to accept SARs in their workplace.	Mixed methods	Intensive care, emergency, medical or surgical, and other units	n=301 nurses	The behavioral intention was moderate with the concerns and benefits of SARs.		Moderate (4)	
Hudson et al (2023) [23], Canada	Perspectives of HCPs in emergency rooms of SARs are designed for IV^d^ insertion distraction.	Qualitative	Emergency room	n=11 (nurses=5, doctors=4, child life specialists=2)	HCPs perceive SARs as a promising tool for distraction during IV insertion.		Moderate (4)	
Kabacińska et al (2025) [28], Canada	Explored the attitudes of HCPs toward the implementation of SARs in a hospital setting.	Multimethods	Pediatrics hospital	n=10 (nurses=5, therapist=4, child life specialist=1)	Reveal potential applications, concerns, and barriers of SARs in a hospital setting.		Moderate (4)	
Kang et al (2023) [32], South Korea	Explored nurses’ perceptions and experiences with SARs in elder care.	Qualitative	Community health settings	n=18 nurses	SARs were useful in caring for older adults with dementia or living alone.		Moderate (4)	
Liang et al (2019) [24], Taiwan	Explored pediatric nurses’ perceptions of SARs, including impacts of clinical work and attitudes.	Qualitative	Pediatric ward	n=23 nurses	The potential of SARs to improve the quality of pediatric care and reduce nurses’ workloads, but some concerns addressed.		Moderate (4)	
Loi et al (2018) [34], Australia	Investigate HCPs’ acceptance of the SARs in engaging residents under 65 years of age.	Cross-sectional	Residential facilities	n=24 nurses	Overall, HCPs were positive about engaging the residents with SARs.		Low (0.5)	
Mlakar et al (2024) [25], Slovenia	Integrating several variables into a comprehensive model that could explain the general acceptance of HCPs to SARs.	Cross-sectional	Regional clinical center	n=490 (nurses=200, doctors=103, others=187)	The acceptance of HCPs was high. The education level and technological expectations influenced the ethical acceptability and acceptance of SARs.		Moderate (3.5)	
Papadopoulos et al (2023) [26], the United Kingdom	Explored global HCPs’ perceived training needs for SARs in their work setting around the world.	Mixed methods	Health and social care sector	n=1284 (nurses=998, midwives=84, others=259)	Knowing the capabilities of SARs was the top training need across countries. Different cultures had different acceptance.		Moderate (4)	
Raigoso et al, (2021) [27], Colombia	Evaluated HCPs’ perceptions of a SARs integrated as part of therapy.	Cross-sectional	Rehabilitation unit	n=77 (therapist=58, psychiatrist=1, others=18)	An overall positive perception of the SARs.		Moderate (3)	
Ramachandran and Lim (2021) [33], Singapore	Investigating the design for a SARs to perform nursing tasks in hospitals.	Cross-sectional	Operation room	n=60 nurses	Politeness and friendliness will increase the perceived trustworthiness and high receptivity with the SARs.		Low (1.5)	
Rigaud et al (2024) [29], France	Explored needs, expectations, and perceptions of the implementation of SARs.	Qualitative	Nursing home	n=20 (nurses=7, doctors=2, others=11)	Most HCPs perceived SARs as useful for nonintimate tasks. The training and education of robots were essential for successful implementation.		Moderate (4)	

^a^HCP: health care professional.

^b^SAR: socially assistive robot.

^c^PD: Parkinson’s disease.

^d^IV: intravenous.

### Quality Assessment

A total of 12 studies were rated as moderate quality [20,22-32] in Table 1, one study as high quality [21], and 2 studies as low quality [33,34]. A detailed description of the quality appraisal process, including the assessment criteria and individual study ratings, is provided in Table S3 in Multimedia Appendix 1.

The primary sources of bias across the included studies pertained to sampling strategies, representativeness, measurement validity, transparency, and the comprehensiveness of reporting. A high-quality study was distinguished by a large sample size and the use of validated measurement instruments. In contrast, studies rated as low quality were characterized by small sample sizes, reliance on self-developed questionnaires without evidence of validity, and reporting bias (Table S4 in Multimedia Appendix 1).

Most of the studies included in the analysis used convenience sampling, often using relatively small samples sourced from a single institution, which may limit the generalizability of their results. However, 1 cross-national study examined whether cultural differences across countries affected HCPs’ training needs for SARs by gathering data from HCPs in 18 countries, with each country contributing between 40 and 140 participants, thereby facilitating cross-cultural comparison [26]. Measurement bias was also observed, as several studies used self-developed instruments without clear reporting of their reliability or validity [26,27,33].

Concerns regarding response bias were identified, as nonresponse rates were rarely reported; only 1 study explicitly documented a nonresponse rate of 25% [25]. Potential confounding factors, such as demographic variables, were infrequently controlled for in quantitative studies, and all qualitative studies relied on subjective interpretations. Some studies with methodological limitations nonetheless reported statistically significant findings [33,34], indicating that such results should be considered within the context of their study designs, and interpretations should be made cautiously.

### Synthesis

#### Overview

The synthesis of findings from all the included studies was achieved through the integration of both qualitative and quantitative evidence. To ensure clarity in interpretation, the results were systematically organized within the UTAUT framework. It is noteworthy that no findings were identified for the social influence dimension, whereas several findings extending beyond the original UTAUT model were classified as additional aspects.

#### Result of Thematic Analysis

**Figure 2:**
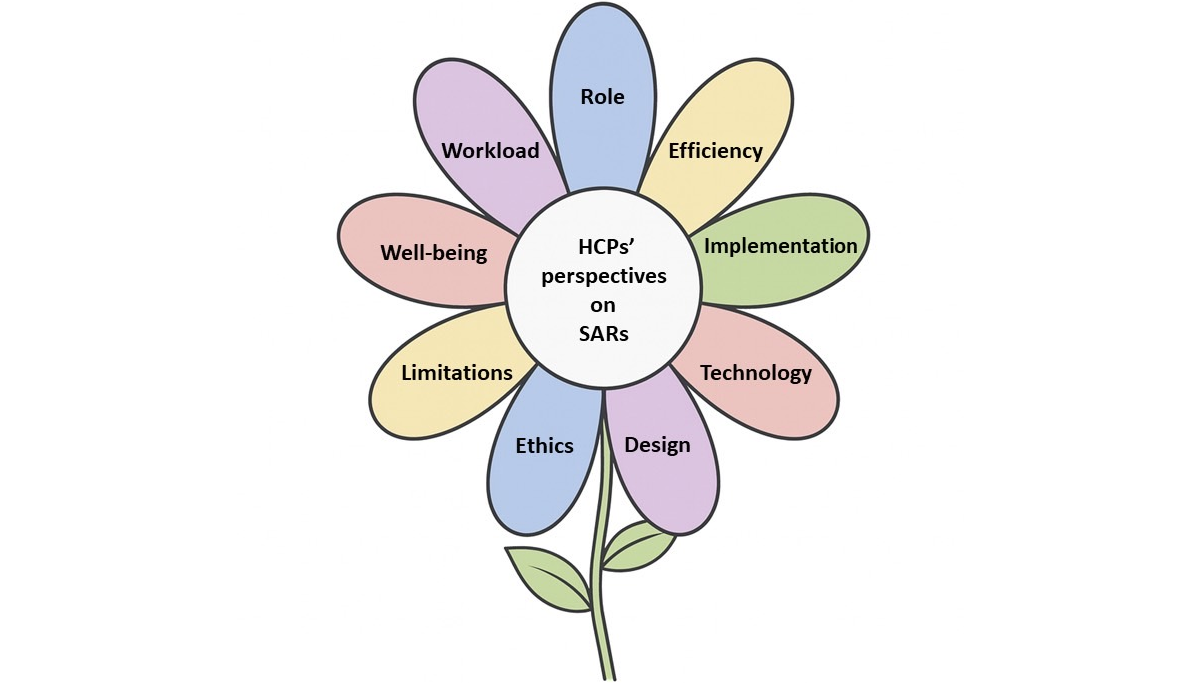
Thematic blossom: health care professional’s (HCP) perspectives on socially assistive robots (SARs).

Thematic analysis of the qualitative data revealed 9 overarching themes (Figure 2) and 25 subcategories (Table 2). To enhance readability, the detailed qualitative data, including full quotes illustrating HCPs’ perceptions, expectations, and concerns regarding the integration of SARs into clinical practice have been illustrated in Table S5 in Multimedia Appendix 1.

The figure illustrates the 9 themes identified through thematic analysis. Petals denote individual themes, with labels derived from concise representations of each theme.

**Table 2:** Results of qualitative findings.

Theme number	Theme	Categories
1	Reducing health care staff workload	Related to professional tasks Related to routine tasks
2	Enhancing care efficiency	Improving care efficacy Enhancing quality of care
3	Promoting patient well-being	Physiological aspects Psychological aspects
4	Limitations of SARs^a^	Lack of emotional perception Inability to replace human presence
5	Technological and operational challenges	Technical malfunction Maintenance and system updates Anticipated barriers during SARs
6	Challenges related to role clarity and professional identity	Additional burden on health care professionals Concerns about being replaced Role definition and responsibility allocation
7	Implementation considerations	Considerations of patient’s physical and psychological safety Environmental requirements Training and educational requirements for SARs Economic cost Implementation strategies Acceptance level
8	Design of SARs	Appearance of SARs Functional capabilities of SARs Mode of operation and user interface
9	Ethical and Influence of SARs Application	Privacy protection Ethical concerns

^a^SAR: socially assistive robot.

#### Integrated Interpretation

##### Overview

The final framework comprises 5 key components: performance expectancy, effort expectancy, facilitating conditions, behavioral intention, and moderating factors. Detailed descriptions of these components are provided below. In addition, several findings that extend beyond the original UTAUT model are categorized as additional aspects. An overview of the framework is presented in Table 3.

**Table 3:** Unified Theory of Acceptance and Use of Technology framework analysis of findings.

Aspect	Definition	Findings
Performance expectancy	The extent to which users believe that using a technology can help them accomplish tasks or enhance performance.	Workload reduction, efficiency, and patient well-being were emphasized.
Effort expectancy	The degree to which users perceive the difficulty of using the technology.	Ease of use, design, and prioritizing training were emphasized, while operational barriers remained critical.
Facilitating conditions	The degree to which users believe that adequate infrastructure and support exist within the organizational and technical environment to facilitate technology use.	Influenced by technical, organizational, and role-related barriers, with limited training, low familiarity, and institutional policies.
Behavior intentions	The extent to which users are willing to use a technology, reflected in acceptance, attitudes, and willingness.	Generally positive attitudes, acceptance, and willingness, influenced by profession and workplace.
Moderate factors	How factors such as sex, age, education level, and experience influence the relationship between the above core constructs and behavioral intention.	The influence of demographic factors, such as age, educational level, sex, and prior use experience, remained inconclusive.

##### Performance Expectancy

Across the studies included, HCPs generally exhibited positive expectations regarding the performance of SARs. Quantitative data indicated that approximately 80% (12/15) of respondents considered SARs to be clinically useful [31]. Additionally, over 58% (14/24) of the participants recognized the potential benefits for residents under the age of 65 years [34]. Although this finding originated from a study with relatively low methodological quality, thereby limiting its credibility, corroborative evidence from other studies supports this perspective. Qualitative findings further emphasized the anticipated benefits of SARs for HCPs, including workload reduction and assistance with routine tasks (Theme 1), enhanced efficiency and quality of care (Theme 2), and promotion of patient well-being (Theme 3). Consistently, an interventional study reported statistically significant improvements following exposure to SARs, with HCPs noting increased comfort in use (*P*=.02) and greater recognition of SARs’ contribution to well-being (*P*=.008) [34]. Nevertheless, this study was appraised as low quality, and the posttest results were based on only 8 respondents, providing insufficient evidence to draw firm conclusions. As a result, interpretations were made cautiously [34].

HCPs have recognized the potential of SARs to enhance care delivery while consistently emphasizing the irreplaceable nature of human attributes such as empathy, compassion, and interpersonal interaction (Theme 4). Consequently, HCPs generally express positive expectations regarding SARs, acknowledging their benefits in reducing workload, improving efficiency, and promoting patient well-being. However, the strength of the evidence was limited by the quality of the studies.

##### Effort Expectancy

Research findings on effort expectancy underscore the pivotal role of ease of use and practical challenges associated with operating SARs in clinical settings. Qualitative investigations further elucidate the barriers perceived by HCPs. Concerns have been articulated regarding operational efficiency, such as the extended time required for device setup and activation, and technical limitations, including the impracticality of frequent charging every 2 hours, which is deemed unsuitable for routine clinical applications (Theme 5). Ease of use and design characteristics have consistently been identified as the critical determinants of adoption. HCPs emphasize the significance of functional capabilities, modes of operation, and user interface design in enhancing usability (Theme 8). Concurrently, unresolved technological and operational issues are noted, including technical malfunctions, maintenance demands, and the requirement for regular system updates (Theme 5). Furthermore, some professionals expressed concerns that the integration of SARs might augment their workload rather than mitigate it (Theme 6).

Training needs are a crucial aspect of effort expectancy. The learning process is often characterized as stressful and daunting, necessitating considerable time and effort for HCPs to acquire the requisite competencies (Theme 7). In a study of moderate quality, “knowing the capabilities of the robot” was identified as the highest training priority, with 43% (n=537) of respondents ranking it as their most important need, followed by “knowing the tasks that the robot can undertake” [26]. This finding is consistent with qualitative evidence, which similarly emphasized that the adequacy of training related to SARs is a key determinant of acceptance (Theme 7). Furthermore, the study investigated demographic variables, including educational level, sex, age, years of work experience, and work setting, in relation to training needs, but no significant differences in the ranking of training needs were observed [26].

These findings underscore the importance of ease of use, design, and usability for SARs adoption, while operational barriers such as time-consuming setups, frequent charging, malfunctions, and maintenance demands were noted. Adequate training is essential for their acceptance.

##### Facilitating Conditions

Research findings suggest that the successful implementation of SARs depends on addressing various contextual and organizational barriers. Key challenges include ensuring patient safety, environmental appropriateness, and managing financial expenditure. Technical issues, including system failures, maintenance requirements, and the necessity for ongoing technical support, were also identified as significant constraints. Furthermore, the lack of familiarity is underscored by the observation that most included studies did not permit HCPs to directly observe or interact with SARs, as findings of moderate factors. In addition, professional differences influenced the perceptions. In a study of moderate quality, reported significant variation between nurses and physicians regarding the perceived capabilities of SARs (*P*=.008) [25], providing evidence of moderate reliability.

In addition to technical and resource-related barriers, concerns about professional identity and role clarity have emerged as significant constraints. There is apprehension that SARs might alter the division of responsibilities within care teams, coupled with uncertainty regarding the allocation of tasks between robots and nursing staff, underscoring the potential for role-related ambiguities (Theme 6). These findings collectively suggest that while HCPs recognize the potential value of SARs, their widespread adoption is impeded by limited exposure, insufficient training, and unresolved technical challenges, as well as by considerations of organizational readiness and professional roles.

Beyond technical and equipment-related challenges, uncertainties regarding role definition and task allocation between HCPs and SARs were also identified. Institutional policies, legal frameworks, and government regulations were deemed critical in determining professional roles and responsibilities.

##### Behavioral Intention

Research findings suggest that HCPs generally hold positive attitudes toward SARs, although acceptance levels vary across different studies and professional groups. In a study of moderate quality, 67.6% (302/447) of HCPs expressed willingness to collaborate with SARs [25]. Over 60% (46/77) of the participants reported favorable acceptance of SARs [27], which is comparable to another moderate-quality study that reported high acceptance mean score of 3.67 (95% CI 3.39-3.94) out of 5 [28]. Additionally, the study found that 84.4% (351/416) of the participants agreed that the use of SARs could make care work more engaging [21].

Notably, professional differences were observed; acceptance levels differed significantly between nurses and physicians (*P*<.001) and between physicians and other professionals (*P*=.03) [25]. Although these findings are rated as of moderate quality, they provide valuable evidence. At the interpersonal level, relational attributes emerged as important determinants of behavioral intention. The perceived politeness of SARs was significantly correlated with friendliness (*r*=0.7) and trustworthiness (*r*=0.81), which were identified as key determinants of behavioral intention and willingness to follow the instructions of SARs [27]. Despite the higher risk of bias in this study, these associations should be interpreted with caution and validated in future studies using more rigorous designs.

Furthermore, a high-quality study reported that attitudes were not correlated with any demographic variables, except for the workplace; participants working in nursing homes had significantly positive (*P*=.02) attitude scores toward SARs [21]. Qualitative findings corroborate these results, with many HCPs expressing favorable views toward SARs (Themes 1-3).

HCPs showed moderate to high levels of acceptance across studies, influenced by profession, workplace, and relational attributes, such as politeness, friendliness, and trustworthiness, although some findings carried higher bias and require cautious interpretation.

##### Moderate Factors

Research findings on the moderating influence of demographic factors have been inconsistent across studies of moderate to high quality, encompassing variables such as sex, age, educational level, and prior experience with SARs.

One study of moderate quality identified sex as significant predictor of acceptance, with male HCPs demonstrating higher levels of acceptance (*P*=.008) [22]. Regarding age, the study reported weak positive correlation with acceptance (*r*=0.14; *P*<.01) [25]; however, subsequent structural equation modeling indicated that age did not significantly predict overall acceptance.

The educational level demonstrated more complex associations. The research reported a positive correlation between higher educational attainment and acceptance (*P*<.001) [22]. Conversely, a high-quality study found no significant effect of educational level on acceptance (*F*=0.17; *P*=.84) [21]. Furthermore, research identified significant association between educational level and both technological capability and ethical acceptability (*P*<.05) [25], but not with overall acceptance. This study also revealed that educational level significantly predicted various dimensions of ethical acceptability, including acceptability of use (β=.20; *P*<.005), interaction with human-like robots (β=.16; *P*<.05), and acceptance of nonhuman appearances (β=.32; *P*<.05). Although these studies offer valuable insights, the effects of sex, age, and educational level on HCPs’ acceptance and ethical acceptability of SARs remain inconsistent.

Quantitative evidence highlights the limited familiarity and experience of HCPs with SARs. For instance, in a high-quality study, nearly half of the respondents (210/416, 50.7%) reported awareness of robots, whereas 49.3% (206/416) had no prior exposure [21].

Evidence on demographic moderators was inconsistent; sex and education were associated with SARs acceptance. Overall, demographic influences remain inconclusive, although education is linked to technological capabilities and ethical acceptability.

##### Additional Aspects

In addition to the primary dimensions of the UTAUT framework, several supplementary factors have been identified. Ethical acceptability has been positively correlated with overall acceptance, with both ethical acceptability for use and ethical acceptability of human-like interaction significantly correlated (*P*<.05) with the general acceptance of SARs [25]. Furthermore, group differences in ethical acceptability were observed, including those between administrators and physicians (*P*<.001), nurses and physicians (*P*<.001), nurses and other professionals (*P*=.01), and physicians and other professionals (*P*=.03) [25]. Although this study was of moderate quality, its findings offer valuable insights into professional variation from an ethical perspective. Qualitative evidence further substantiated these results, underscoring recurring ethical concerns. These concerns encompassed the protection of patient privacy; safeguarding of informed consent and autonomy; and assurance of equity of access, justice, and fairness (Theme 9). HCPs also emphasized the necessity of establishing clear boundaries between human and robotic roles, expressing apprehensions about robots potentially overstepping professional responsibilities, or inappropriately recording sensitive information (Theme 9).

Consequently, ethical acceptance was positively correlated with acceptance, with significant group differences across professions. Concerns focused on privacy, informed consent, equity, and clear role boundaries, highlighting ethics as a key supplementary factor influencing implementation.

## Discussion

### Overview

This systematic review investigates HCPs’ perspectives on the clinical application of SARs. This study synthesizes key issues, including the challenges and barriers faced by HCPs in using SARs, their levels of acceptance, influencing factors, ethical literacy, educational needs, and considerations for clinical implementation. Additionally, this review offers recommendations for the implementation of SARs.

### Challenges and Barriers for HCPs in Using SARs

This review highlights the diverse challenges faced by HCPs when integrating SARs into clinical practice, with technological reliability standing out as a central concern. Commonly reported issues include hardware malfunctions, software errors, limited battery capacity, and unstable connectivity. Such problems can interrupt clinical workflows and undermine HCPs’ trust in SARs. These findings echo Masala and Giorgi [35], who demonstrated a strong link between perceived reliability and trust.

Beyond technical limitations, concerns also extend to SARs’ ability to interpret emotional expressions and nonverbal cues. Many HCPs perceive these systems as lacking empathy and emotional intelligence qualities essential for person-centered care. While acknowledging that SARs can provide valuable support for routine tasks, HCPs remain doubtful about their ability to preserve the interpersonal aspects of caregiving, a concern similarly raised by Zuschnegg et al [36]. Respondents also questioned whether SARs could demonstrate sound judgment and adaptability in complex clinical situations.

Professional identity represents another source of tension. Some nursing staff expressed concern that robots might intrude on core nursing responsibilities, potentially threatening their professional roles. This ambivalence reveals a paradox; on one hand, HCPs emphasize the irreplaceable value of human caregiving, and on the other, they anticipate institutional pressures to replace human labor with robotic alternatives. Importantly, such perceptions may be shaped less by direct experience with SARs and more by media portrayals or imagination.

Overall, it suggests that HCPs tend to view SARs as complementary tools rather than substitutes, consistent with the findings of Madi et al [37]. However, research results presented a counterpoint, arguing that SARs may fail to reduce workload and could even add to it, due to the resources needed for implementation and maintenance [38]. This raises concerns about their long-term sustainability. Nonetheless, in the context of workforce shortages, SARs could still play a meaningful role by alleviating routine burdens, provided that future designs emphasize intuitive human-robot interaction (HRI) and user-friendly functionality. Such improvements would ensure that SARs are seen as practical assistants that enhance, rather than hinder, clinical practice.

This review also found that the role of education in shaping HCPs’ acceptance of SARs varied across studies. These inconsistencies may stem from differences in study quality, regional context, or the professional backgrounds of participants. Interestingly, age did not appear to have a significant influence, which contrasts with the UTAUT, where age is considered a key moderating factor. This discrepancy may be explained by HCPs’ prior experience with technology. This conclusion is supported by the research findings of Naneva and colleagues [39], who determined that familiarity with similar technologies enhances the willingness to adopt SARs.

Although education level and age are often viewed as important moderators in UTAUT, current evidence on their impact remains inconclusive. Future research should use more rigorous methods to clarify the role of these variables in shaping HCPs’ acceptance of SARs.

### Ethical Literacy

The reviewed studies indicate that HCPs express concerns regarding “blurred boundaries” and “compromised patient dignity” in HRI, although empirical evidence in this domain remains limited. The specific challenges identified include the absence of adequate regulations for AI-driven decision-making, risks associated with algorithmic bias, and issues pertaining to data privacy. For instance, under the General Data Protection Regulation, cross-border transmission of patient data necessitates explicit consent and encryption. Furthermore, if the training data are not representative, algorithmic bias may ensue, leading to AI recommendations that disadvantage specific groups [38]. These concerns are consistent with broader calls for robust ethical guidelines and governance frameworks to ensure the “responsible adoption” of AI in clinical settings [40-42].

Building on these perspectives, this review underscores the necessity for ethical frameworks governing SARs to integrate the principles of transparency, accountability, and inclusivity throughout the design, development, and clinical deployment of AI systems. The incorporation of ethical literacy serves as both a professional competency for HCPs and an institutional safeguard, thereby mitigating potential risks and fostering responsible innovation. This argument aligns with the conclusions of El Arab et al [43] and Zhang et al [44]. Practical strategies may involve the establishment of “robot ethics committees” by convening HCPs, relevant experts in ethics and law, and technology developers within hospitals to oversee data use, informed consent processes, and cultural sensitivity in HRI. At the policy level, governments might consider forming national “AI ethics committees” to regulate technological trajectories and amend existing laws as necessary, as also proposed by Zhang et al [44].

Notably, Mlakar et al [25] identified a positive correlation between ethical acceptability and overall acceptance of SARs by HCPs, highlighting the potential influence of ethics on adoption decisions. Collectively, these findings indicated that ethical literacy directly affects clinical integration. Consequently, future research should aim to validate the extent to which enhancing HCPs’ ethical literacy facilitates the adoption and sustainable use of SARs.

### Educational Needs

**Figure 3:**
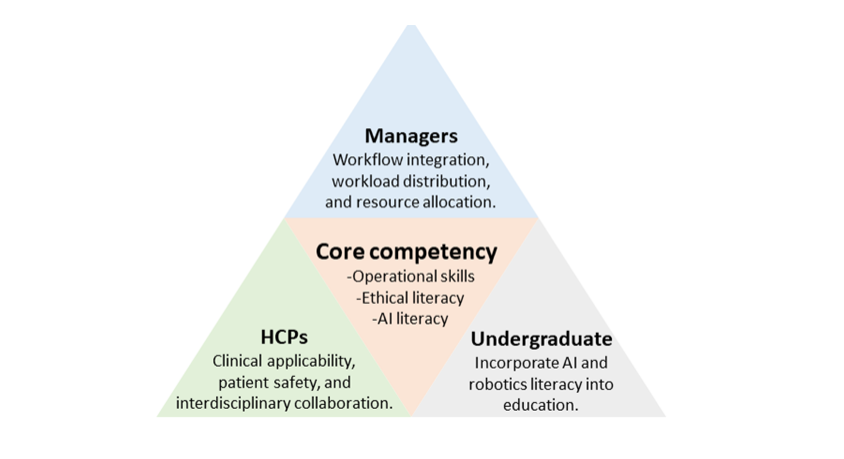
Core competencies and educational needs in health care. AI: artificial intelligence; HCP: health care professional.

Beyond individual attitudes, organizational support for education and resource investment is crucial in influencing HCPs’ acceptance and adoption of SARs (Figure 3). The reviewed studies consistently demonstrate that successful clinical integration necessitates comprehensive training programs that encompass operational skills, AI literacy, and ethical literacy. Among these, proficiency in daily operations, such as process flow, troubleshooting, and maintenance, has emerged as the most immediate requirement. A deficiency in these competencies may lead to clinical setbacks and diminished willingness to engage. This finding aligns with that of Langer et al [45], who emphasized that understanding both the functions and limitations of SARs is a key prerequisite for effective implementation.

This review emphasizes the necessity of integrating ethical literacy as a core component of SARs training, addressing patient autonomy, data privacy, informed consent, and the equilibrium between efficiency and empathy in care. Enhancing these competencies can assist HCPs in forming realistic expectations and reducing their frustration in clinical applications. The study further contended that as AI and robotics increasingly influence patient safety, quality of care, ethical decision-making, data privacy, and resource efficiency, training requirements must extend beyond basic technological literacy [46]. Therefore, it is recommended to incorporate AI and robotics literacy into training curricula, starting with undergraduate and prelicensure education. The research endorsed this approach, asserting that such measures are essential for future HCPs to effectively interpret AI outputs and apply them in practice [43].

Despite these varied needs, most institutions currently lack formal training programs dedicated to SARs, with learning often occurring informally through peer-to-peer exchange. This reliance risks neglecting essential competencies in the technical, ethical, and HRI domains. Addressing these gaps requires the development of flexible, interdisciplinary training initiatives or guiding principles that can be adapted to local contexts. Such initiatives would enhance professional preparedness for SARs integration while remaining aligned with the overarching goal of delivering safe, efficient, and person-centered care [43].

Training requirements differ significantly across professional roles, highlighting the need for customized approaches. Nursing professionals often prioritize practical operational competencies such as process flow, troubleshooting, and patient-facing interaction skills. Conversely, managers tend to focus on workflow integration, workload distribution, and resource allocation. Meanwhile, physicians and rehabilitation specialists should concentrate on clinical applicability, patient safety, and interdisciplinary collaboration. Consequently, training programs should be modular and tailored to specific roles, ensuring that each professional group acquires the competencies most relevant to its responsibilities. This reliance may result in the neglect of competencies in the technical, ethical, and HRI domains, highlighting the urgent need for standardized, interdisciplinary training initiatives supported by institutional policies and practice guidelines [43].

It is imperative to enhance the early integration of technological literacy into health care education. AI has the potential to revolutionize professional learning and augment digital health competencies among prospective clinical staff members. Therefore, structured curricula should emphasize foundational AI concepts, ethical principles, and simulation-based training. Educators should also be trained to ensure the effective incorporation of AI into both clinical and educational settings [43].

The triangular framework illustrates the interrelated educational needs of managers, HCPs, and undergraduates. These include operational skills, AI literacy, and ethical literacy, which are essential for the responsible integration of AI and robotics into health care practice.

### Practical Implementation

The successful integration of SARs into clinical practice necessitates consideration of both environmental preparedness and organizational support. Infrastructure readiness is essential, and clinical environments must be modified to accommodate SARs through suitable physical layouts, charging facilities, and docking stations. Engaging HCPs in the co-design process can enhance feasibility, promote ownership, and increase acceptance. Implementation may also benefit from phased incremental strategies, starting with pilot projects, and providing continuous feedback to ensure sustainability and adaptability. A clear delineation of roles and responsibilities is crucial, specifying which tasks SARs should perform and which remain within the purview of HCPs. This aligns with Elendu et al [47], who emphasized transparent decision-making, clear attribution of responsibility, and structured task allocation.

In addition to workflow integration, economic feasibility and policy endorsements are critical determinants. Conducting cost-effectiveness analyses is essential to assess procurement, training, maintenance, and infrastructure in relation to anticipated benefits such as improved efficiency, reduced workload, and enhanced patient outcomes. These considerations align with the findings of Gkiolnta et al [38], who emphasize that although HCPs acknowledge the potential value of SARs, their adoption ultimately hinges on practical viability and institutional commitment.

### Directions for Future Research

This review highlights several deficiencies in the extant literature concerning HCPs’ perspectives on SARs. Primarily, existing studies predominantly emphasize short-term outcomes, with insufficient exploration of long-term experiences such as user satisfaction and sustained engagement. Given that many HCPs lack direct interactions with SARs, research frequently depends on video demonstrations or hypothetical scenarios. Consequently, future research should prioritize field trials within authentic clinical settings to produce evidence that more accurately reflects real-world practice.

Second, the propensity to adopt SARs is anticipated to differ among various professional roles and clinical environments, underscoring the need for comparative studies of distinct groups of HCPs. Further research is imperative to evaluate the technical reliability and adaptability of SARs in the medical setting. Interdisciplinary collaboration is crucial, and joint training initiatives between HCPs and robotics engineers may facilitate the alignment of technological designs with clinical requirements. Ethical considerations constitute another significant area of research, necessitating the development of concrete ethical assessment tools and practical guidelines to guide value-driven decision-making.

Interdisciplinary collaboration is essential for the effective integration of technologies, as evidenced by this review that advocates the establishment of joint training programs between HCPs and robotics engineers. Such initiatives aim to foster mutual understanding of clinical needs and technological constraints, thereby enhancing the feasibility of applications. Furthermore, ethical considerations require further exploration, particularly in the development of practical assessment tools and context-sensitive guidelines to support value-driven clinical decision-making.

Hence, cross-national variation is evident in how SARs are perceived and adopted, reflecting differences in health care systems, professional norms, and cultural attitudes toward technology [13]. These factors influence not only levels of acceptance but also the type and intensity of training required. Accordingly, while this synthesis consolidates global evidence, its generalizability remains constrained, and future research should prioritize culturally sensitive approaches to inform policy and practice.

Further research should investigate the impact of “robot literacy” on HCPs’ acceptance and implementation outcomes. At the educational level, previous literature has suggested that medical and nursing schools could explore integrating courses on “AI and robotics applications,” ideally in collaboration with AI and engineering faculties, to help upcoming HCPs develop essential technological competencies. Rather than prescribing rigid, standardized curricula, such training has been proposed in the form of adaptable modules or guiding principles tailored to various professional roles, clinical contexts, and cultural environments. Exploring these approaches in future studies may help address existing knowledge gaps and foster collaboration among HCPs, data scientists, ethicists, and engineers, thereby supporting the co-design of ethical, person-centered, and clinically relevant AI systems [43].

Although UTAUT offered a strong analytical structure, several themes in this review—ethical considerations, professional identity, and organizational readiness—extend beyond its original constructs. These findings suggest that UTAUT may be insufficient on its own to fully capture the complexities of SARs adoption in health care. Future research should consider adapting UTAUT or developing an expanded model that incorporates these dimensions, drawing on complementary theories from implementation science and ethics. By identifying these extensions, this review not only applies UTAUT but also contributes to refining its applicability in health care contexts.

### Strengths and Limitations

This review had several strengths. By systematically synthesizing evidence from the perspective of HCPs, this study underscores an essential yet frequently underexplored dimension of SARs—the factors influencing HCPs’ acceptance and adoption. This focus is particularly significant as HCPs’ engagement is a critical determinant of whether SARs can be effectively integrated into clinical practice. This review provides a comprehensive account of HCPs’ perceptions, expectations, and concerns by integrating qualitative, quantitative, and mixed methods studies across diverse professional backgrounds and clinical contexts.

The application of the UTAUT as an analytical framework further enhances the interpretive coherence and theoretical grounding of the findings. Collectively, these features augment the relevance and forward-looking value of this review, providing insights that can guide both technological design and clinical implementation strategies.

Several limitations should be noted. A limited number of multimethod studies failed to clearly articulate their methodologies, and qualitative and quantitative findings were reported separately, thereby hindering their integration. In addition, qualitative studies often rely on small sample sizes, which may limit representativeness, while numerous quantitative studies use questionnaires lacking fully validated reliability or validity, potentially compromising explanatory power. In mixed methods research, relatively few studies have concentrated exclusively on HCPs; in several instances, quantitative data were gathered from patients or family members, with HCPs’ perspectives primarily represented through qualitative evidence.

The heterogeneity of study designs and outcomes not only complicated subgroup analyses but also precluded the possibility of conducting a meta-analysis, thereby limiting the strength of quantitative synthesis. Additionally, some studies included only nursing staff, whereas others did not provide detailed information on professional backgrounds, complicating comparisons across roles or disciplines.

Moreover, inconsistencies in quantitative instruments and a lack of comparable data render meta-analysis impractical. Collectively, these limitations underscore areas in which future research could enhance the quality and comparability of evidence. Nonetheless, by consolidating diverse findings and emphasizing the pivotal role of HCPs, this review makes a timely and significant contribution to the understanding of how SARs may be effectively integrated into health care, offering forward-looking insights for research, policy, and practice.

One key limitation is that many included studies involved HCPs without direct experience using SARs. This restricts ecological validity, as the findings largely represent anticipated perceptions rather than insights grounded in real-world interaction.

Another limitation is the quality of the included studies. Among the 15 studies synthesized, only 1 was rated as high quality [21], 2 were low quality [33,34], and the remainder as moderate quality. As a result, our interpretations were made cautiously to avoid overestimating the impact of SARs.

Despite these limitations, this review underscores the centrality of HCPs’ perspectives in shaping the future of SARs and informs the development of policies, training initiatives, and design strategies that can promote responsible and sustainable integration.

### Conclusion

This review highlights the pivotal role of HCPs in shaping the effective integration of SARs into clinical practice. Across diverse contexts, HCPs acknowledged the potential of SARs to reduce workloads, improve efficiency, and support patient well-being, while also raising concerns about technological reliability, ethical challenges, and professional boundaries. Ethical literacy and targeted training programs have been discussed as potential enablers of acceptance, underscoring the importance of comprehensive, interdisciplinary approaches that incorporate transparency, accountability, and person-centered care principles.

In addition, methodological inconsistencies were evident, and most of the included studies were of only moderate quality, with limited high-quality evidence. Small sample sizes and the use of nonvalidated instruments further reduce generalizability. Consequently, our interpretations were intentionally cautious to avoid overstating the potential impact of SARs, and the actual readiness of HCPs for adoption remains uncertain. The successful clinical implementation of SARs also depends on broader contextual factors such as organizational readiness, economic feasibility, and clear task delineation to ensure sustainability and trust. While these considerations extend beyond the direct findings of this review, they align with themes highlighted in previous literature and represent important directions for further inquiry. Despite the limitations of the current evidence base, this review contributes a theoretically grounded synthesis using the UTAUT framework and consolidates diverse perspectives of HCPs. Future research should prioritize longitudinal and context-specific evaluations, engage participants with direct SARs experience, and foster interdisciplinary collaboration to generate stronger evidence for the responsible, equitable, and sustainable adoption of SARs in health care.

## Appendix

Multimedia Appendix 1 Full search strategies across the database, excluded articles, and the reasons and risks of bias of included studies.

Multimedia Appendix 2 PRISMA checklist.

## Abbreviations

AI: artificial intelligence
HCP: health care professional
HRI: human-robot interaction
MeSH: Medical Subject Headings
MMAT: Mixed Methods Appraisal Tool
PICO: Population, Intervention, Comparison, and Outcome
PRISMA: Preferred Reporting Items for Systematic Reviews and Meta-Analyses
PROSPERO: International Prospective Register of Systematic Reviews
SAR: socially assistive robot
UTAUT: Unified Theory of Acceptance and Use of Technology
