# Supplementary material for: Health Care Professionals’ Perspectives of Socially Assistive Robots in Health Care Settings: Systematic Review
Source: J Med Internet Res. 2025 Oct 9;27:e79634. doi: 10.2196/79634 (PMC12550450; doi:10.2196/79634)
Supplement: Multimedia Appendix 1 [file jmir_v27i1e79634_app1.docx]

# **Table S1.** Full search strategies across the database.

| Name of Database | Number of search results | Search strategies  (including Boolean operators, filters, and limits) |
| --- | --- | --- |
| PubMed (MEDLINE) | 77 | ("Healthcare Professionals"[MeSH] OR "Nurses"[MeSH] OR "Physicians"[MeSH] OR "Medical Staff" OR "Clinicians")  AND ("Socially Assistive Robot"[MeSH] OR "SAR" OR "Healthcare Robot" OR "Assistive Robotics")  AND ("Acceptance"[MeSH] OR "Attitude" OR "Perception" OR "Perspectives" OR "Views") AND (clinical OR "Healthcare Setting" OR "Patient Care") |
| Cochrane Library | 242 | (Healthcare Professionals OR Nurses OR Physicians OR Medical Staff OR Clinicians)  AND (Socially Assistive Robot OR SAR OR Healthcare Robot OR Assistive Robotics)  AND (Acceptance OR Attitude OR Perception OR Perspectives OR Views) AND (clinical OR Healthcare Setting OR Patient Care). Choose Title、Abstract、Keyword |
| Scopus | 28 | ("Socially Assistive Robot"[MeSH] OR "SAR" OR "Healthcare Robot" OR "Assistive Robotics")  AND ("Acceptance"[MeSH] OR "Attitude" OR "Perception" OR "Perspectives" OR "Views"). Choose keyword, title, abstract |
| IEEE Xplore | 31 | (Healthcare Professionals OR Nurses OR Physicians OR Medical Staff OR Clinicians) AND (Socially Assistive Robot OR SAR OR Healthcare Robot OR Assistive Robotics) AND (Acceptance OR Attitude OR Perception OR Perspectives OR Views). In metadata |
| ScienceDirect | 30 | TITLE ("Healthcare Professionals" OR "Nurses" OR "Physicians" OR "Medical Staff" OR "Clinicians")  AND TITLE-ABSTR-KEY("Socially Assistive Robot" OR "SAR" OR "Healthcare Robot" OR "Assistive Robotics")  AND TITLE-ABSTR-KEY (Acceptance OR Attitude OR Perception OR Perspectives OR Views) |
| CINAHL with Full Text (EBSCO) | 13 | ("Healthcare Professionals" OR "Nurses" OR "Physicians" OR "Medical Staff" OR "Clinicians") AND ("Socially Assistive Robot" OR "SAR" OR "Healthcare Robot" OR "Assistive Robotics") AND (Acceptance OR Attitude OR Perception OR Perspectives OR Views) |
| Epistemonikos | 4 | ("Healthcare Professionals" OR "Nurses" OR "Physicians" OR "Medical Staff" OR "Clinicians") AND ("Socially Assistive Robot" OR "SAR" OR "Healthcare Robot" OR "Assistive Robotics") AND (Acceptance OR Attitude OR Perception OR Perspectives OR Views) |
| MEDLINE (OVID) | 21 | ("Healthcare Professionals" OR "Nurses" OR "Physicians" OR "Medical Staff" OR "Clinicians") AND ("Socially Assistive Robot" OR "SAR" OR "Healthcare Robot" OR "Assistive Robotics") AND (Acceptance OR Attitude OR Perception OR Perspectives OR Views). |
| Web of Science (WOS) | 60 | (Healthcare Professionals OR Nurses OR Physicians OR Medical Staff OR Clinicians) AND (Socially Assistive Robot OR SAR OR Healthcare Robot OR Assistive Robotics) AND (Acceptance OR Attitude OR Perception OR Perspectives OR Views) AND (clinical OR Healthcare Setting OR Patient Care). Abstract. |
| Embase | 10 | (Healthcare Professionals OR Nurses OR Physicians OR Medical Staff OR Clinicians) AND (Socially Assistive Robot OR SAR OR Healthcare Robot OR Assistive Robotics) AND (Acceptance OR Attitude OR Perception OR Perspectives OR Views) AND (clinical OR Healthcare Setting OR Patient Care) |
| UpToDate | 0 | Technology acceptance by healthcare professionals, Socially assistive robots in healthcare settings. Acceptance OR Attitude OR Perception OR Perspectives OR Views |

# **Table S2.** Excluded articles and the reasons.

| No. | Excluded articles | Exclude reasons |
| --- | --- | --- |
| 1 | Healthcare robots and human generations: Consequences for nursing and healthcare | Opinion article |
| 2 | Evaluation of nurses’ perspectives on the design and use of assistant nurse robots in obstetrics and neonatal care: a mixed-method study | Not focused on SARs |
| 3 | An User-Centered Evaluation of Two Socially Assistive Robots Integrated in a Retirement Home | Not involving HCP participants |
| 4 | Social robots in adult psychiatry: a summary of utilisation and impact | Not involving HCP participants |
| 5 | Technological Innovation Adoption Among Swedish Healthcare Professionals: A Contingency Technology Adoption Framework | Not focused on SARs |
| 6 | Has Telemedicine come to Fruition? Patients’ and Physicians’ Perceptions Regarding Telemedicine: Presenter(s): Noelle Junod Perron, Geneva University Hospitals and Geneva Faculty of Medicine, Switzerland | Not focused on SARs |
| 7 | Navigating artificial intelligence in care homes: Competing stakeholder views of trust and logics of care | Not focused on SARs |
| 8 | Longitudinal changes following the introduction of socially assistive robots in nursing homes: a qualitative study with ICF framework and causal loop diagramming | No relevant outcomes reported |
| 9 | Perceived Usefulness of a Social Robot Augmented Telehealth Platform by Therapists in the United States | Not involving HCP participants |
| 10 | Robots and Robotics in Nursing | Opinion article |
| 11 | Finnish healthcare professionals' attitudes towards robots: Reflections on a population sample | Not focused on SARs |
| 12 | Role of Robotics and Artificial Intelligence in Oral Health and Preventive Dentistry - Knowledge, Perception and Attitude of Dentists | Not focused on SARs |
| 13 | Development and usability evaluation of a bedside robot system for inpatients | Not focused on SARs |
| 14 | Becevic(2015)Robotic Telepresence in a Medical Intensive Care Unit Clinicians’ Perceptions | Not focused on SARs |
| 15 | Papadopoulos et al. (2022). Socially assistive robots in health and social care: Acceptance and cultural factors. Results from an exploratory international online survey | Overlapping dataset, retained the more relevant article. |

**Table S3.** Quality Assessment of Included Studies.

| **Author**  **(year)** | **Question 1** | **Question2** | **Question3** | **Question4** | **Question5** | **Overall**  **(Score)** |
| --- | --- | --- | --- | --- | --- | --- |
| Bar-on and Mayo (2023) [20] | Y^a^/Y^a^/Y^a^/Y^a^/Y^a^ | NA^c^ | NA^c^ | NA^c^ | NA^c^ | Moderate  (4) |
| Bradwell et al.(2021) [30] | Y^a^/UC^d^/Y^a^/  Y^a^/Y^a^ | NA^c^ | NA^c^ | NA^c^ | NA^c^ | Moderate  (3) |
| Casas et al. (2019) [31] | Y^a^/Y^a^/UC^d^/  UC^d^/Y^a^ | NA^c^ | NA^c^ | Y^a^/UC^d^/Y^a^  /UC^d^/Y^a^ | NA^c^ | Moderate  (3) |
| Chen et al. (2020) [21] | NA^c^ | NA^c^ | NA^c^ | Y^a^/Y^a^/Y^a^/  UC^d^/Y^a^ | NA^c^ | High  (4.5) |
| El-Gazar et al. (2024) [22] | Y^a^/Y^a^/Y^a^/Y^a^/Y^a^ | NA^c^ | NA^c^ | Y^a^/Y^a^/Y^a^/Y^a^/Y^a^ | Y^a^/Y^a^/UC^d^/  N^b^/Y^a^ | Moderate  (4) |
| Hudson et al. (2023) [23] | Y^a^/Y^a^/Y^a^/Y^a^/Y^a^ | NA^c^ | NA^c^ | NA^c^ | NA^c^ | Moderate  (4) |
| Kabaci ´ nska et al. (2025) [28] | Y^a^/Y^a^/Y^a^/Y^a^/Y^a^ | NA^c^ | NA^c^ | Y^a^/Y^a^/Y^a^/Y^a^/Y^a^ | NA^c^ | Moderate  (4) |
| Kang et al. (2023) [32] | Y^a^/Y^a^/Y^a^/Y^a^/Y^a^ | NA^c^ | NA^c^ | NA^c^ | NA^c^ | Moderate  (4) |
| Liang et al. (2019) [24] | Y^a^/Y^a^/Y^a^/Y^a^/Y^a^ | NA^c^ | NA^c^ | NA^c^ | NA^c^ | Moderate  (4) |
| Loi et al. (2018) [34] | NA^c^ | NA^c^ | N^b^/Y^a^/N^b^/N^b^/UC^d^ | NA^c^ | NA^c^ | Low  (0.5) |
| Mlakar et al. (2024) [25] | NA^c^ | NA^c^ | NA^c^ | Y^a^/Y^a^/Y^a^/  UC^d^/Y^a^ | NA^c^ | Moderate  (3.5) |
| Papadopoulos et al. (2023) [26] | Y^a^/Y^a^/Y^a^/Y^a^/Y^a^ | NA^c^ | NA^c^ | Y^a^/Y^a^/Y^a^/Y^a^/Y^a^ | Y^a^/Y^a^/Y^a^/Y^a^/Y^a^ | Moderate  (4) |
| Raigoso et al. (2021) [27] | NA^c^ | NA^c^ | NA^c^ | Y^a^/Y^a^/UC^d^/  UC^d^/Y^a^ | NA^c^ | Moderate  (3) |
| Ramachandran et al. (2021) [33] | NA^c^ | NA^c^ | NA^c^ | Y^a^/Y^a^/UC^d^/  UC^d^/UC^d^ | NA^c^ | Low  (1.5) |
| Rigaud et al. (2024) [29] | Y^a^/Y^a^/Y^a^/Y^a^/Y^a^ | NA^c^ | NA^c^ | NA^c^ | NA^c^ | Moderate  (4) |

^a^Y: Yes

^b^N: No

^c^NA: Not Appropriate

^d^UC: Unclear

# **Table S4.** Risk of Bias of Included Studies.

| Author  (year) | Study design | Risk of Bias | Overall |
| --- | --- | --- | --- |
| Bar-on and Mayo (2023) | Qualitative Research | Convenience sampling and limited reporting transparency; measurement tools were not validated. | (Moderate) |
| Bradwell et al.(2021) | Qualitative Research | Convenience sampling with data analysis heavily reliant on the researcher's interpretation; measurement tools were not validated. | (Moderate) |
| Chen et al. (2019) | Quantitative descriptive | Convenience sampling with no reporting of non-response rates. | (low) |
| Hudson et al. (2023) | Qualitative Research | Small sample size, single-country setting. | (Moderate) |
| Kang et al. (2023) | Qualitative Research | Convenience sampling with samples heavily concentrated among female dementia-care nurses, further influenced by the COVID-19 context. | (Moderate) |
| Liang et al. (2019) | Qualitative Research | Sample composition (all female, single-country setting) and potential bias from voluntary participation. | (Moderate) |
| Loi et al. (2018) | Non-randomized quantitative | High attrition rates, failure to account for confounders, and potential deviations from the intended intervention protocol. | (Moderate) |
| Mlakar et al. (2024) | Quantitative descriptive | Convenience sampling subject to self-selection bias, with only interested individuals participating; non-response rate of 25%. The instrument was originally designed for children. | (Moderate) |
| Raigoso et al. (2021) | Quantitative descriptive | Convenience sampling with unknown refusal/non-response rates; instruments were theoretically informed but self-developed, with validity unreported until post-hoc. | (Moderate) |
| Ramachandran et al. (2021) | Quantitative descriptive | Convenience sampling with unknown refusal/non-response rates; self-developed questionnaires without validation; analyses relied on correlations despite small sample sizes and unreported potential confounders. | (high) |
| Rigaud et al.(2024) | Qualitative Research | Small sample size, convenience sampling, gender imbalance, and regional limitations restricting external validity. | (Moderate) |
| Casas et al. (2019) | Multi-method | Convenience sampling, small sample, lack of demographic controls, and leading questions in open-ended survey items. | (Moderate) |
| El-Gazar et al.(2024) | Mix-method | Quantitative component demonstrated methodological rigor (large sample, validated tools, high response rate); however, the qualitative component was limited by small sample size and unvalidated instruments. | (Moderate) |
| Kabaci ´ nska et al. (2025) | Multi-method | Inclusion of diverse populations and multi-method data collection (surveys and focus groups) with tools largely adapted from established scales; however, limited by convenience sampling, small sample sizes, language barriers, and demographic skew. | (Moderate) |
| Papadopoulos et al. (2023) | Mix-method | Reliance on convenience sampling, use of self-developed questionnaires, unknown national response rates, and potential semantic issues arising from cultural translation. | (Moderate) |

# **Table S5.** Theme analysis of qualitative findings.

| *Theme 1: Reducing healthcare staff workload* | |
| --- | --- |
| Definition: This theme highlighted the potential of SARs to undertake repetitive, time-consuming, and standardized tasks, thereby supporting healthcare professionals in focusing on more complex responsibilities and alleviating stress related to time and workload pressures. | |
| Categories | Quotations |
| *1.1*  *Related to professional tasks* | “Robotics can help me to prepare the precise medicine for pediatric patients, including injections and oral administration, even very tiny doses. Pediatric patients are tiny, not like adults. I have to watch out that I am administering the precise dose.” [24]  “It is convenient to be able to monitor the client’s activities and manage them over the phone without the need for physical contact.” [32]  “Given our limited time, the robot could offer companionship, engaging in conversations residents are eager to have […]” [29] |
| *1.2*  *Related to routine tasks* | “Robotics can assist in introducing the setting for children and caregivers consistent. Videotaped instruction presented by robots would allow caregivers to return to material they have questions about and rewatch that specific segment of the video. Robots could save nurses a lot of time.” [24]  “Automating tedious tasks would not only free up our time but could also alleviate the stress of our daily workload, fostering a more relaxed environment.” (Rigaud et al., 2024)  “Robots could alleviate physical strain by performing repetitive or physically demanding tasks.” [22] |
| *Theme 2: Enhancing care efficiency* | |
| Definition: SARs were perceived as tools that could streamline clinical workflows, lessen staff burden, and concurrently improve patient comfort, engagement, and therapeutic support—fostering care that is both efficient and high in quality. | |
| *2.1 Improving care efficacy* | “Robotics can allow family members to feel assured by immediate access to care counseling and guidance, and confident about their preparedness for occasional abnormal events.” [24]  “Ensuring that in the event of a fall, the robot can immediately alert staff, enhancing its practicality and effectiveness.” [29]  “It is convenient to be able to monitor the client’s activities and manage them over the phone without the need for physical contact.” [32] |
| *2.2 Enhancing quality of care* | “They’re kind of like part of the care team right? So, if they’re being asked questions about how they’re feeling, or what are they doing, that it’s not just between the parent or caregiver and the health care team. It’s involving the child themselves.” [28]  “And then, [to use it] for added practice, as the thing that connects the clinic to the home environment, and that brings something [new] into the routine, and enables to practice the technique we learned in the therapy session in different environments, that would be amazing.” [20]  “Given our limited time, the robot could offer companionship, engaging in conversations residents are eager to have […].” [29] |
| *Theme 3: Promoting patient well-being* | |
| Definition: This theme explored SARs’ contributions to patients’ physical and psychological well-being through companionship, cognitive stimulation, and emotional support, ultimately enhancing the overall care experience. | |
| *3.1 Physiological aspects* | “the physical therapist can use it to help the patient practice walking and balance, so you take the [robotic] dog for a walk, and you enjoy that too.” [20]  “the robot can favor the stimulation of cognitive functions of the residents, such as memory and language and help the involvement of the person in the activity by encouraging the user to imitate it (the robot).” [29]  “The pain of an injection is also perceived by how the kid’s feeling, with anxiety and stress, and that’s more painful. So, I think that the outcome, potentially your hypothesis, is that if the child’s feeling better, then the pain is perceived less.” [28] |
| *3.2 Psychological aspects* | “There’s something about pets and animals, even ones that are not real but look like they’re real. They feel like they’re interacting with them, even if they’re sleeping. It’s really company for most of the kids.” [28]  “During COVID-19, Aria played a big role in emotionally supporting older adults.” [32]  “I think cute robots could make my patients smile or decrease their anxiety instead of seeing me in the nurse uniform with injection equipment most of the time. I believe robotics is a good buffer in the relationship between patients and me. They can comfort the children before I come.” [24] |
| *Theme 4: Limitations of SARs* | |
| Definition: Concerns were raised regarding SARs’ limited ability to perceive emotions, interpret nonverbal cues, or engage in natural human interactions. These limitations prompted skepticism about their capacity to replicate genuine human connection and empathy, as well as the risk of overreliance on robotic systems. | |
| *4.1*  *Lack of emotional perception* | “It is hard for a robot to assess patients' mood and feelings and respond appropriately.” [24]  “Robots have no sense to feel children's anxiety, fear, happiness, or sadness. How can they offer adequate care for children and their families?” [24]  “I don’t know what the robots kind of reactions are like based on reading faces. But if there was a potential that it, it could read the room wrong. I mean, give an inappropriate reaction, or even just be scary for a little one.” [28] |
| *4.2*  *Inability to replace human presence* | “It cannot replace human communication... it can never be like a real human being. [20]  “Robots cannot provide the empathetic and compassionate care that human nurses offer. ”[22]  “It’s crucial that the personal touch in caregiving is preserved; robots can’t replace that.” [29]  “It is okay to use [a robot] as a tool to help the patient, but not in a way that replaces other social needs, such as meeting their family members.” [20] |
| *Theme 5: Technological and operational challenges* | |
| Definition: This theme encompassed concerns related to system malfunctions, maintenance demands, connectivity issues, and charging limitations that may compromise the reliability and consistency of SARs in clinical environments. | |
| *5.1*  *Technical malfunction* | “One of the problems... when it breaks down or power outage […]” [20]  “Sensors could fail in the measurements and report wrong data.” [31]  “One of the problems when you depend on technology is what happens when it breaks down, when there is a power outage, or when the battery runs out. […] Therefore, a non-electrical alternative or a backup is needed. When you depend on it, what happens when it does not work?” [20] |
| *5.2 Maintenance and system updates* | “The ability to have ongoing [...] updates of if it was going to actually say things, [...] would like to say ‘Count to 3’, and then they’re going to do the injection.” [28]  “Potential for robots to malfunction, require maintenance and disrupting care.” [22]  “The tasks include receiving the device when someone returns it, replacing it with another when the device breaks down, teaching and answering questions, reconnecting it again when Bluetooth is not working, and so on.” [32] |
| *5.3*  *Anticipated barriers during SARs* | “I think those would be my biggest barriers to using it—efficiency. [...]. We’re getting a lot of pressure to get surgery done, there’s a big wait list like. So, like if I’m sitting there trying to get Wi-fi for like 15 min, like someone’s going to just tell me to stop or something.” [28]  “it might indeed replace social connections. Beyond the connection with the formal caregiver, the patient may reduce their other social connections because they’re enjoying the time they spend with the robot, [...]” [20]  “I’m a little worried on that, that it might become an extra thing of noise that can’t be stopped.” [23] |
| *Theme 6: Challenges related to role clarity and professional identity* | |
| Definition: This theme encompasses concerns related to system malfunctions, maintenance demands, connectivity issues, and charging limitations that may compromise the reliability and consistency of SARs in clinical environments. | |
| *6.1 Additional burden on healthcare professionals* | “It can’t be a half an hour we have to push the robot out of the closet and turn it on and it has to load. That’s where you lose time.” [23]  “Constantly needing to recharge the battery every 2 hours would be impractical for us.” [29]  “How long does it take [to] learn, […] oh it’s a bit too scary [...], do you need quite a lot of training?” [30] |
| *6.2*  *Concerns about being replaced* | “I am worried that robots might take over our jobs. It’s not just about the tasks they can perform, but also about how our roles will change. Will there still be a place for us, or will we be sidelined.” [22]  “Maybe it’s like, oh, we don’t. We don’t need child life. We have the robot because they can also prepare—like it’s just like a very different skill set. But yeah, I feel like that might be a potential barrier from [the] team.” [28]  “If robots help us to do the clinical tasks, we'll reduce nurses' clinical skills and maybe lose our jobs.” [24] |
| *6.3*  *Role definition and responsibility allocation* | “They’re kind of like part of the care team right? So, if they’re being asked questions about how they’re feeling, or what are they doing that it’s not just between the parent or caregiver and the health care team. It’s involving the child themselves. ” [28]  “There must be a line between the robot and ourselves... the robot should not overstep human roles or record private information.” [29]  “There is uncertainty about how the roles of robots and human nurses will be defined and managed.” [22] |
| *Theme 7: Implementation considerations* | |
| Definition: Key factors for successful SARs adoption included patient safety, environmental suitability, staff training, financial costs, and user acceptance—highlighting the need for phased, context-sensitive implementation strategies. | |
| *7.1*  *Considerations of patient physical and psychological safety* | “I decided for the ranking because I would really like to know what a robot can do and how to program it so that it does not hurt the patient but performs the activities as safely as possible.” [26]  “Before the robots' administering of medication, we would have to check to see if the robot is functioning normally or not. It may be out of order. If we don't check, it will harm pediatric patients.” [24]  “There’s a delicate balance in ensuring activities facilitated by the robot are within the president’s ability range to prevent feelings of inadequacy.” [29] |
| *7.2*  *Environmental requirements* | “The robot requires clear pathways, but obstacles like armchairs and other frequently used items in the facility can impede movement.” [29]  “The patient spends most of his time at home, right? We need to help him at home, in his personal environment, to help him with his everyday needs.” [20]  “Biggest barriers... trying to get Wi-fi […].” [28] |
| *7.3 Training and educational requirements for SARs* | “I am interested in the capabilities, benefits and operation of the robot in order to know how to use it optimally for the patient. Additional training in a simulator to achieve competence in operating the robot and prevent damage to the patient.” [26]  “Continuing education in knowledge and clinical skills, as well as in advanced technology such as the function and usage of robots, is important for nurses to respond to pediatric patients' physical, mental, and social care needs.” [24]  “Training is essential; without proper introduction and ongoing support, these technologies can become more of a burden than a help.” [29] |
| *7.4*   \| *Economic*  *cost* \| \| --- \| | “These services are free (to selected people) because we are paying for them. By increasing government support to lowering the burden, when a household with older adults (with dementia) apply for a service directly (not through a public center), this (care robot) will be more widely used.” [32]  “The financial outlay for a robot encompasses not only its purchase price but also ongoing maintenance, necessary institutional modifications for deployment, insurance, and further maintenance costs. These aspects require thorough financial assessment.” [29]  “I could see cost being a barrier. Just the fact that there are so many different places in this hospital that a robot could be used that there’s...it would take a lot of money for every place to have a robot for everyone to see.” [28] |
| *7.5*  *Implementation strategies* | “We should start with trials to see how well these robots integrate into our care routines.” [29]  “And because we’re so used to doing IVs without a robot, people will very quickly notice if there’s any significant barriers to making it happen. So, I think it’s cool [to have] the opportunity to see how it works and once it’s there, the workflow needs to be as seamless as possible without barriers. Because if you put up barriers, people abandon it very quickly.” [23]  “The issue of liability, especially if a robot were to cause any harm, necessitates careful legal scrutiny. It appears that legal experts are beginning to explore these complex questions.” [29] |
| *7.6*  *Acceptance level* | “I believe robotics is a good buffer in the relationship between patients and me. They can comfort the children before I come.” [24]  “We can do whatever they want to do and then why we should have them?” [26]  “just with everything we’ve done—been over the last 3 years, so it’s hard to bring new things in and bring change, and it’s like we’ve had so much change over the last 3 years. It’s like “not another thing.” [28] |
| *Theme 8: Design of SARs* | |
| Definition: This theme emphasized the significance of SARs’ physical appearance, functional capabilities, and user-friendly interfaces in promoting clinical usability and fostering acceptance among healthcare staff and patients. | |
| *8.1*  *Appearance of SARs* | “A humanoid size would facilitate collaborative interaction.” [29]  “The robot can be designed to have a human-like conversation, though obviously it is not a replacement for a human. So, for people whose default state [is reclusion], the robot could improve their situation.” [20]  “change how hard it is, like if it was softer.” [30] |
| *8.2 Functional capabilities of SARs* | “A lot of the things that have been mentioned, the playing games, or the engagement, or even the distraction piece of it, tell me that would all be applicable for communication.” [28]  “I hope robots can help us track the result of patients' lab data and interpret it, since patients always have multiple tests in the lab and we get the data back at different times. If robots can collect all results and interpret blood tests, genetic tests, and other tests for us, it will help a lot […].” [24]  “…If they’re [referring to the AI-enhanced SARs] able to pick up and read off their emotions, I could see that as being super helpful and then you don’t direct the robot at all, if they’re able to see that the child is scared, and then change their approach based on that.” [23] |
| *8.3 Mode of operation and user interface* | “Many things (functions) have to go through multiple paths (steps). Educating older adults on the route for each function is not easy. For example, if (someone) presses and holds number 1 or 2 on the cell phone, it automatically connects to your child’s cell phone. Like that, it must be simple and easy to operate.” [32]  “I have reservations about robots that utilize simplistic or juvenile interfaces, as it’s crucial to maintain respect and not diminish the adults we’re serving.” [29] |
| *Theme 9: Ethical and Influence of SARs Application* | |
| Definition: Ethical concerns centered on patient privacy, data security, informed consent, and equitable access, underscoring the necessity for robust ethical frameworks to guide the responsible deployment of SARs in clinical settings. | |
| *9.1*  *Privacy protection* | “Are they catching things in the background? (...) Even if the parents are okay with it, are they catching other things that they shouldn’t be.” [28]  “If everyone could easily get patients' data, or change data, it would harm patients and their personal privacy. Furthermore, it is hard for robots to maintain privacy in a public area. Everyone there could hear the robots' answers.” [24]  “And the risk of the robot being hijacked? The threat of cyber-attacks remains a real and present danger.” [29] |
| *9.2*  *Ethical concerns* | “A practice guide could be formulated to ensure ethical interactions by the robot, including seeking consent, adapting its communication style to the resident’s preferences, respecting privacy, and ensuring equitable engagement across the resident population.” [29]  “When I was thinking equitability, […] sometimes, when we have certain resources that there’s not that many of. And if there’s a number of us that are all wanting to use it at the same time, how do you decide which clinicians going to have the robot, to use the things.” [28]  “For example, an occupational therapist can be replaced with an app. The patient will receive treatment, but it will be of lower quality, less tailored, and may even [...] cause harm. But it will be much cheaper, much simpler, and much more accessible.” [20] |
